# Induced Changes in Aroma Compounds of Foods Treated with High Hydrostatic Pressure: A Review

**DOI:** 10.3390/foods10040878

**Published:** 2021-04-16

**Authors:** Adrián Lomelí-Martín, Luz María Martínez, Jorge Welti-Chanes, Zamantha Escobedo-Avellaneda

**Affiliations:** Escuela de Ingeniería y Ciencias, Tecnologico de Monterrey, Eugenio Garza Sada 2501, Monterrey, NL 64700, Mexico; alomeli@exatec.tec.mx (A.L.-M.); luzvidea@tec.mx (L.M.M.); jwelti@tec.mx (J.W.-C.)

**Keywords:** aroma compounds, high hydrostatic pressure, lipid-derived aroma compounds

## Abstract

Since conventional thermal processing can have detrimental consequences on aroma compounds, non-thermal technologies such as high hydrostatic pressure (HHP) have been explored. HHP may alter the weak chemical bonds of enzymes. These changes can modify the secondary, tertiary, and quaternary structures of key enzymes in the production of aroma compounds. This can result in either an increase or decrease in their content, along with reactions or physical processes associated with a reduction of molecular volume. This article provides a comprehensive review of HHP treatment’s effects on the content of lipid-derived aroma compounds, aldehydes, alcohols, ketones, esters, lactones, terpenes, and phenols, on various food matrices of vegetable and animal origin. The content of aldehydes and ketones in food samples increased when subjected to HHP, while the content of alcohols and phenols decreased, probably due to oxidative processes. Both ester and lactone concentrations appeared to decline due to hydrolysis reactions. There is no clear tendency regarding terpenes concentration when subjected to HHP treatments. Because of the various effects of HHP on aroma compounds, an area of opportunity arises to carry out future studies that allow optimizing and controlling the effect.

## 1. Introduction

Odor, mostly known as the sense of smell, can be defined as the process undertaken by the olfactory system in which volatile compounds going through the nasal membranes provoke a stimulus as a response, while aroma is the odor an object possesses [1]. These volatile compounds responsible for an object’s aroma are called aroma compounds. Although the concentration of aroma substances present in food matrices is extremely low, around 10–15 mg/kg [2], the number of components is astonishingly high; coffee, for example, has around 1000 identified volatile organic compounds [3]. Among all the different aroma substances that can be identified in food matrices, special attention is given to compounds linked to the characteristic aroma of the food and are, consequently, called “*character impact compounds*” [2]. One of the most important characteristics an aroma compound can have is its odor threshold, the lowest concentration of a compound possible that allows recognition of its odor; these data enable comparisons of the intensity or potency between compounds of the same matrix to be made [4]. 

Alongside taste, food aroma is the main attribute controlling a consumer’s choice of a product [1]. In a market flooded with competition, the conservation of these compounds is a priority in the food processing industry to sway consumers’ choices. Due to the potential loss in total aroma compounds that results from traditional thermal treatments, the current focus of the industry lies in the optimization of conventional processing as well as the development of novel technologies. One such novel non-thermal technology is high hydrostatic pressure (HHP) that generates safe and stable food products but with the advantage of not damaging the sensory or nutritional properties of the product [5,6]. 

HHP technology involves using isostatic pressures between 100 and 1000 MPa [7]. HHP processing does not require an active source of heating. However, increases and decreases in the temperature of the subjected products exist due to adiabatic compressions and decompressions, respectively. Since this increase in temperature does not exceed 50 °C when water is used as a pressure transiting medium, HHP is categorized as a non-thermal process due to the passive nature of the heating it provokes [8]. HHP processing may present both desired and undesired effects in the matrices being subjected to treatment. An example of the positive effects HHP treatments may have is in cellular wall and membrane disruption, eliminating pathogenic and deteriorative microorganisms to ensure food safety and extend the product’s shelf-life [9,10]. On the other hand, an example of a possible negative effect HHP has in food matrices is altering the secondary, tertiary, and quaternary structures of enzymes responsible for desired compounds [11,12] due to the breakage of weak interactions, mainly hydrophobic and electrostatic interactions [13], thus changing the concentration of the compounds of interest. 

The present contribution intends to overview the most relevant findings, specifically on how HHP processing affects the concentration of the main types of volatile aroma compounds in food matrices. The performance of HHP treatments is compared to traditional thermal processing methods when applicable to gauge if HHP can be an alternative in the commercial food processing industry in terms of retaining the aroma profile of foods. 

## 2. Aroma Compounds in Foods

Aroma can have several functions in food besides stimulating the appetite; it can also convey essential characteristics of the item by alerting when a food item is rancid or unsafe to consume [14]. Chemical compounds responsible for the aroma in food products are highly volatile, and they are found at very low concentration. Aroma properties can be influenced by factors such as carbon chain length, presence or absence of double bonds, and aromatic rings. Some of the most current and studied species found in food matrices are the lipid-derived aroma compounds, namely aldehydes, alcohols, ketones, esters, lactones, terpenes, and phenols [15], which are explored in the following paragraphs (Table 1).

### 2.1. Aldehydes 

Aldehydes are organic compounds containing a terminal carbonyl functional group (“-CHO”), they are extremely common components of any food or flavoring agent, and many possess a low odor threshold [16]. Acetaldehyde, for example, is a crucial component of many fruit aroma profiles imparting fruity ether notes [17,18,19], whereas the C_3_–C_5_ aldehydes, propanal, butanal, and pentanal, tend to possess a rather chemical/malty/green note to them that is hard to define [20,21]. As the chain length increases beyond C_6_, the aldehydes take on a dual character and have both fruity/floral and fatty descriptors to their aroma, depending on the concentration present, the matrix, and the individual who is perceiving them [22]. 

Unsaturated aldehydes, which can exist in both (E) and (Z) isomers conformations, tend to have higher odor thresholds and are often character impact compounds, with the shorter chain analogs providing green aromas [23,24]. It is also worth mentioning that the odor threshold for both saturated aliphatic aldehydes and unsaturated aldehydes seems to decrease progressively with the increase in chain length [23].

Aldehydes containing an aromatic ring such as benzaldehyde, phenylacetaldehyde, and cinnamaldehyde are all important components of foods and flavorings [14] which dominantly present sweet, nutty aromas such as that of freshly crushed almonds [20]. 

### 2.2. Alcohols

Alcohols are organic compounds that possess at least one hydroxyl (“-OH”) group and are also abundant in foods and flavorings, but their contribution to aroma tends to be significantly less than their corresponding aldehydes due to the considerably higher odor threshold alcohols possess [25]. 

Straight-chain alcohols are abundant in fruits; they often increase with maturity due to the decrease in the carbonyl content produced by the enzymatic breakdown of unsaturated fatty acids [26]. The terpene alcohol linalool is an extremely important compound in the aroma profile of both citrus fruit and spices contributing in large part to the distinctly fresh floral and fruity notes of oranges and lemongrass [26,27]. Geraniol is considered another essential terpene alcohol due to its contribution to the characteristic floral and fruity notes of fruits such as lychee [27,28]. Although both linalool and geraniol are very important components of the aroma profiles in the matrices they are found in, this behavior is mainly due to them being terpenes rather than typical alcohol behavior. 

Introducing a double bond into the chain reduces odor threshold when compared to other alcohols and makes them character impact compounds with earthy and musty tones, such as in the case of mushrooms [14].

### 2.3. Ketones

Ketones are organic compounds containing a carbonyl center functional group “RC(=O)R”. Most ketones possess a relatively high odor threshold when compared to other lipid-derived aroma compounds such as aldehydes or esters [14]. However, they are still important components to both dairy products and fruit flavor profiles [15,27].

Straight-chain methyl ketones, containing one carbonyl group in the 2-position such as 2-heptanone, 2-nonanone, and 2-undecanone impart both blue cheese and a fruity pear aroma depending on the matrix it is encountered [29,30]. 

The α-dicarbonyl compounds, such as 2,3-butanedione, 2,3-pentanedione, 2,3-hexanedione, and 2,3-heptanedione, have far lower thresholds than other species of ketones and are responsible for buttery and/or creamy notes in many cooked foods or flavorings [31,32]. Ketones derived from carotenoids such as α-ionone, β-ionone, and β-damascenone can provide both pippy notes in orchard fruits and deep juicy notes in, for example, berries, tomatoes, and apples [27].

### 2.4. Esters

Esters are acid-derived compounds in which at least one of the structure’s hydroxyl (“OH”) groups is substituted by an “O-R” group, where R symbolizes a carbon-containing substituent. Esters are fundamental to the aroma of most fruits, comprising the major proportion of the volatile compounds in, for example, melons, apples, pineapple, and strawberries [33,34,35,36]. Ethyl esters are major components of fruit aroma, particularly of ripe fruit, where ethanol production boosts their formation [14]. Some species of esters can be quite characteristic of specific fruits: ethyl 2-methylbutanoate is characteristic of pear or pear drops [37], both methyl and ethyl hexanoate are typically found in pineapple [35], cis-3-hexenyl butanoate imparts the green leafy aroma of the parent alcohol [14], and C_9_ esters are important components of melon aroma [33]. 

### 2.5. Lactones

Lactones are cyclic or intramolecular esters (“R−C(=O)−O−R”) formed by the intermolecular esterification of the corresponding hydroxy acid. Due to most lactones possessing low odor thresholds, they are considered potent aroma compounds [28]. It is also worth mentioning that the odor thresholds of lactones tend to decrease significantly as the number of constituent carbons increases [14]. Most δ-lactones are found in animal products, whereas γ-lactones preferentially occur in plants [38,39]. The δ-lactones, which are based on a pyran ring, are less odor-active than their furanyl isomers [25] and impart distinct sweet odors in dairy products such as butter and milk. Those based on a furan ring are γ-lactones and tend to impart peachy, creamy, and coconut aromas [14]. 

### 2.6. Terpenes 

Terpenes are a type of hydrocarbon, major components of essential oils, and responsible for the characteristic aroma profile of many fruits, particularly citrus fruits such as orange and lemon [27]. They are biosynthesized in plants from units of isoprene (C_5_H_10_) and can be linear, cyclic, or polycyclic; however, those that are responsible for odor tend to contain two or three isoprene units (monoterpenes and sesquiterpenes, respectively) [14]. Many terpenes can be readily oxygenated, although, technically, these are terpenoids and not terpenes. One of the most ubiquitous is limonene, which has a weak orangey citrus peel aroma, but it is not a powerful odorant. Citral is popular in the flavor industry and exists as a mixture of the (E) and (Z) isomers, which are called geranial and neral, respectively [15].

### 2.7. Phenols 

Phenols are a class of organic aroma compounds in which at least one hydroxyl group (“OH”) is directly bonded with an aromatic hydrocarbon ring. Many phenols are particularly odor-active compounds. The methylphenols are particularly phenolic and smoky [40]. It is also worth mentioning that more complex phenols have been identified and presumed as character impact compounds in foods and beverages with more desirable aroma characteristics than their simpler counterparts [14]. The guaiacols or methoxyphenols are described with many terms; 4-methylguaiacol is described as sweet, candy, vanilla, leather, spicy, and smoky [41], whereas 4-ethylguaiacol and 4-vinylguaiacol have a similar range of descriptors but also include a meaty bacon character [42]. Many character-impact compounds of spices are phenols, e.g. eugenol from cloves (both an alcohol and a phenol) and vanillin from vanilla (both an aldehyde and a phenol) [14].

**Table 1:** Lipid-derived aroma compounds.

Compound		Aroma	Matrix	Reference
Aldehydes	Nonanal	Fruity	Plum	[43]
	Propanal	Malty	Bread	[44]
	Butanal	Green	Carrot	[14]
Alcohols	Gerianol	Floral	Lychee	[14]
	Cis-3-hexen-1-ol	Green	Grape	[2]
	Geosmin	Musty	Mushroom	[15]
Ketones	β-Damascenone	Fruity	Berries	[26]
	2,3-Heptanodione	Buttery	Cereals	[45]
	2,3-Butanodione	Creamy	Cookies	[46]
Esters	Ethyl Hexanoate	Fruity	Pineapple	[27]
	Cis-3-Hexenyl Butanoate	Green	Passion Fruit	[47]
	Phenethyl Acetate	Sweet	Grape Juice	[26]
Lactones	6-Hexalactone	Sweet	Almond	[2]
	6-Decalactone	Peachy	Peach	[47]
	γ-Butyrolactone	Creamy	Coconut	[14]
Terpenes	Citral	Citric	Orange	[27]
	β-Damascenone	Malty	Wheat Honey	[44]
	Citronerol	Green	Wine Grape	[14]
Phenols	Vanillin	Sweet	Vanilla	[2]
	Methyl-Eugenol	Spicy	Spicy Beef	[14]
	4-Vinylguaiacol	Smoky	Bacon	[45]

### 2.8. Synthesis and Chemical Changes of Aroma Compounds in Food

Volatile aroma compounds in food are primarily synthesized by one of three types of processes [48]:

1. Enzymatic and microbial processes liberate low molecular weight volatiles. These types of processes are particularly important in the aroma of fruits and vegetables. An example of this is vanillin, the main aroma component of vanilla extract [49].
2. Chemical precursors are produced during a fermentation step. Character impact compounds in both cocoa (phenylacetaldehyde) [50] and bread (1,2-propanedione) [51] are generated by the heating of precursors formed by these types of reactions.
3. Non-enzymatic processes result from thermal treatments, such as pasteurization, sterilization, cooking, and roasting [52,53]. These reactions typically refer to the decomposition of lipids, carbohydrates, and proteins.

Out of the three processes described above, enzymatic and non-enzymatic ones are of particular interest in the food industry due to their roles in two of the major routes to flavor and aroma profile formation and alteration, lipid oxidation and the Maillard reaction, respectively [45]. 

Lipid oxidation refers to the process that involves the degradation of fatty acids, where hydrogen is abstracted from a lipid to leave a lipid radical, which in turn reacts with molecular oxygen to form a lipid peroxide. In this process, unsaturated fatty acids form stabler radicals when compared to saturated fatty acids, mainly due to conjugation of the radical with the double bonds, hence being more prone to oxidation even at lower temperatures. Saturated fatty acid degradation becomes more important at higher temperatures, such as during thermal processing [45,54]. Lipid oxidation primarily forms short-chain aldehydes, alcohols, and ketones [55,56], but the formation of esters and phenols is also possible via a photo-oxidation mechanism [54].

The Maillard reaction is a chemical reaction between amino acids and reducing sugars often also referred to as non-enzymatic browning. From a structural chemistry perspective, the Maillard reaction can be divided into early stages and late stages, where further reactions require more control due to their capacity of forming potentially harmful compounds such as acrylamide, heterocyclic aromatic amines, and advanced glycation end-products [57,58]. Aroma-responsible compounds formed via Maillard reactions are divided into three categories: oxygen-containing molecules (aldehydes, ketones, and furanones), nitrogen-containing molecules (pyrazines and pyrrolines), and sulfur-containing compounds molecules (thiazoles and thiazolines, dithiazines, furanthiols, and sulfides) [46,59,60]. 

While other reactions such as caramelization (enols and lactones) [61,62], and both thiamine (sulfur compounds) [63] and ferulic acid degradation (phenols) [64] are other sources of important aroma compounds, experts in the food industry tend to focus on lipid oxidation and the Maillard reaction due to the sheer number of compounds found to be produced by both mechanisms [45,57]. 

Food processing is a necessary step to ensure microbiological safety and shelf-life requirements to be met, as well as the generation of multiple volatile aroma compounds characteristic of the matrix they are found in [65]. Although thermal processes are effective mechanisms for microbial inactivation, they are also responsible for physical and chemical changes in the product’s sensory qualities [66]. Among these changes are changes in texture because of the effects that heat has on the product’s surface, color because of the deconstruction of color due to non-enzymatic browning, nutritional quality due to the loss of thermosensitive nutriments, and aroma due to the generation of off-odor compounds [5,6,63,67]. To avoid these undesired side-effects, the food industry aims to develop non-thermal technologies that ensure food safety and sensory qualities, one such technology being high hydrostatic pressure processing.

## 3. HHP Effect on Aroma Compounds of Foods

Emerging as an alternative to thermal treatments, HHP process is a food preservation method in which the product is subjected to pressures in the range of 100–1000 MPa to inactivate microorganisms and enzymes [9,68,69,70]. Because this process is carried out at a temperature of ~4–50 °C, it is considered as a non-thermal technology. In high pressure technology, the sample, which can be either solid or liquid, is placed in a chamber filled with a liquid (most commonly water) which acts as the pressure transmitting medium. The almost instantaneous and uniform effect of the application of HHP (isostatic principle) facilitates the scaling of processes from laboratory level to industrial level, thus representing an important commercial advantage of this technology [71]. Samples placed in the treatment chamber are pressurized either by pumping the medium in the chamber (indirect pressurization) or by reducing the chamber volume using a piston (direct pressurization).

The effect that HHP has in structural and/or functional properties of the microorganisms, chemical reactions, phase changes, and other products to be pressurized is governed mainly by two principles: the isostatic and the Le Chatelier–Braun principles [72]. Due to the isostatic principle, pressure is uniformly transmitted regardless of the composition, size, and shape of the food as well as equipment [73]. The Le Chatelier–Braun principle states that, for any change in pressure (P) in a system at constant temperature (T), variations in the volume and energy of this same system will occur and their magnitudes will directly depend on both the levels of the applied variables and the physicochemical properties of the system (such as compressibility). In this way, the activation volume (V_a_) is a parameter derived from the dependence of the pressure with the reaction rate constant (*k*). This volume is defined as the difference between the molar partial volume of the transition state and that of the reactants and whose value is determined by evaluating the effect of pressure at constant temperature on k [74].

Lnk = lnk_0_ − [(V_a_ ∗ P)/(R ∗ T)] (1)

In Equation (1), if V_a_ is negative, the reaction will be favored by pressure (P), and, if it is positive, the opposite will occur. The higher is the absolute value of V_a_, the greater is the sensitivity of the reaction to pressure, while reactions with V_a_ = 0 are independent of pressure [75]. Therefore, the effect of pressure on V_a_ will be a decrease, increase or no change if V_a_ < 0, V_a_ > 0, or V_a_ = 0, respectively.

**Figure 1:**
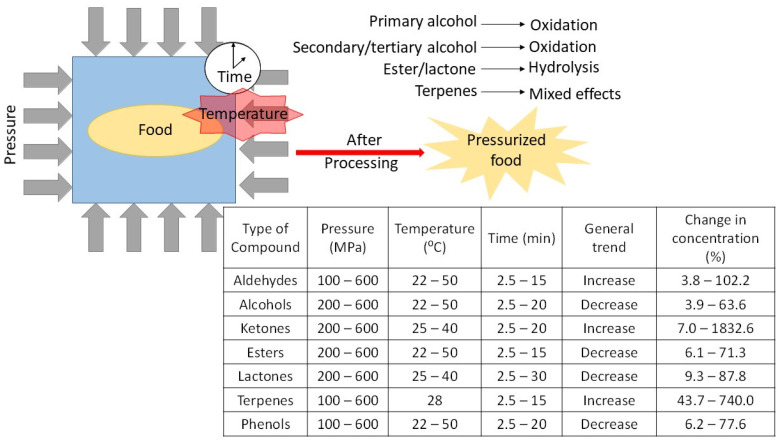
General effects of HHP on aroma compounds of foods.

HHP has minimal effects on the nutritional quality of foods due to the fact that these levels of pressure are not capable of altering covalent bonds up until pressure ranges of 1000–2000 MPa [44,76,77]; therefore, the structures of aroma compounds and primary protein structures will not be affected. It is worth mentioning, however, that pressure does affect intermolecular interactions such as hydrogen bonds and electrostatic interactions [76]; this results in an impact on the secondary, tertiary, and quaternary structure of proteins, thus explaining the inactivation of microorganisms as well as enzymes [76,78,79]. Since the overall content of volatile compounds in matrices has been previously linked to enzymatic reactions [12,69,70], this effect, along with the fact that pressure favors reactions in which the molecular volume of the system decreases, serves to explain the possible change in concentration of aroma compounds in treated samples. Until now, it has not been possible to accurately predict whether HHP has a positive, negative, or negligible effect on the volatile compounds of systems, and, consequently, individual investigations are required for each reaction and/or food matrix that is of interest [74]. However, certain tendencies regarding volatile aroma compounds have been identified from previous works (Figure 1) and are discussed in the following sections. 

### 3.1. HHP Effect on Aldehydes and Alcohols

An overview of the main changes in the concentration of aldehydes and alcohols after HHP treatments at 100–600 MPa is shown in Table 2. With a few notable exceptions that are discussed in a further paragraph, most food matrices seemed to have an increase in the total content of aldehydes (3–261%) and a decrease in the content of alcohols (4.1–90.9%). This increase can be attributed to the oxidation of free fatty acids, such as linoleic and linolenic acids present in most of the referenced matrices, which probably occurred during storage after HHP due to enzymatic activity [43,80,81,82].

Fatty acids are converted into hydroperoxides, which in turn are easily converted into a C_6_ aldehyde through cleavage by a hydroperoxide lyase, resulting in the formation of compounds such as (Z)-3-hexenal, (E)-2-hexenal, and n-hexanal. An enhancement on this reaction due to HHP processing can be observed in kiwifruit pulp [81], mulberry juice [83], and green asparagus juice [84] where the content of 2-hexenal was enhanced when compared to control samples, albeit presenting a maximum concentration at different processing conditions. On average, the processing conditions to maximize the production of aldehydes oscillates within 200–400 MPa depending on the matrix [43,83]. As previously mentioned, not all matrices present an increase in the concentration of aldehyde content, as is the case where C_9_ aldehydes such as (E)-2-nonenal and (E,Z)-2,6-nonadienal are predominant as in Keitt mango juice [85]. These aldehydes are products of the peroxidation of polyunsaturated fatty acids, a reaction catalyzed by lipoxygenase, an enzyme that has previously been found to be inactivated in most HHP treatment conditions [85,86]. Another example of a notable decrease in the content of aldehydes can be seen on Mulberry juice [83], where, although the overall content of aldehydes was enhanced after HHP processing, (E)-2-Heptenal, benzaldehyde, and (E)-2-Nonenal concentrations decreased significantly (around 42.6–82.1%). With the exception of Mulberry juice [83], the total content of alcohols in HHP-treated samples is reduced significantly, which can be mainly attributed to oxidation of alcohols after treatments, thereby producing aldehydes and offering yet another explanation for the overall increase of aldehydes. 

Hexanol is an important aroma compound in fruit and vegetable matrices, generally providing grassy and floral notes [14]. It is obtained from hexanal via the catalysis of alcohol oxidoreductase found in plant tissue, therefore an increase in hexanol levels can be an indication of enhanced oxidoreductase activity produced by HHP. In red plum puree [43], the levels of hexanol were similar to unprocessed purees, which suggests that HHP did not affect the oxidoreductase activity. However, this is not the case for all other matrices; in kiwifruit pulp, it was noted to decrease with both HHP and thermal treatments [81], while, in mulberry juice HHP treatments at 500 MPa, it slightly increased hexanol concentration [83]. HHP treatments maintained an overall higher aldehyde concentration when compared to traditional thermal treatment, likely due the additional enzymatic oxidation processes favored by the pressure. It is also worth mentioning that, despite alcohol concentration being lower than in untreated samples, HHP treatments maintained a higher concentration of them when compared to thermal processing, which is attributed to the sensitivity of these compounds to thermal treatment.

**Table 2:** Main changes in the concentration of aldehydes and alcohols after HHP treatments.

Matrix	Processing Conditions	Storage Conditions and Technique of Analyses	Main Results	Reference
**Aldehydes**				
Red Plum Puree	400 and 600 MPa/1 s, 2.5–5 min	Samples were stored at −80 °C for one week before volatile analysis via headspace SPME-GC	11 Aldehydes were found in the samples: acetaldehyde, 2-methylbutanal, pentanal, hexanal, (2z·4e)-hexa-2.4-dienal, hex-2-enal, heptanal, (e)-oct-2-enal, nonanal, decanal, and dodecanal.	[43]
			The total AAU of the aldehydes isolated was slightly increased after HHP (↑ 3.8–7.2%), except in purees processed at the less intense conditions (400 MPa/1 s) which showed similar total amounts as unprocessed purees (↓ 0.8%) The highest increase of aldehydes was found in purees processed at 400 MPa/5 min (↑7.2%)	
			Hexanal was not affected by HHP, being the major aldehyde andrepresenting more than the 75% of the total area of aldehydes	
Kiwifruit Pulp	400 and 600 MPa/5–15 min	Samples were stored in the dark at 4 °C for 40 days before volatile analysis via SPME and GC-MS	14 Aldehydes were identified in the samples: 3-hexenal, hexanal, (E)-2-hexenal, Nonanal, 2-propenal, butanal, 2-Butenal, Pentanal, 3-methyl-Butanal, (E)-2-decenal, octanal, (E,E)-2,4-heptadien-al, 2-decenal, and 2-undecenal	[81]
			The levels of most aldehydes such as hexanal, (E)-2-hexenal, nonanal as well as (E, E)-2,4-heptadienal increased with HHP treatment (↑ 5–102.2%)	
			The highest increase of aldehydes content was observed in samples treated at 500 MPa for 10min (↑ 8.7–102.2%)	
Keitt Mango Juice	200, 400, and 600 MPa/15 min	Samples were frozen in liquid nitrogen after processing and stored at −80 °C for two weeks until the volatile analysis via GC-MS	4 aldehydes were identified in the samples: (E)-2-heptenal, 1-nonanal, (E)-2-nonenal, and (E,Z)-2,6-nonadienal	[85]
			C_9_ aldehydes ((E)-2-nonenal and (E,Z)-2,6-nonadienal) seemed to decrease with increase of processing pressure	
			(E,Z)-2,6-non-adienal and (E,Z)-3,6-nonadien-1-ol were found to be significantly lower as the pressure reached above 400 MPa	
Germinated Brown Rice (GBR)	100, 300, and 500 MPa/15 min	Samples were stored at 4 °C for 36 h until the volatile analysis via GC-MS	20 aldehydes were identified in the samples: methacrolein, 2-methyl-propanal, butanal, 2-methyl-butanal, 3-methyl-butanal, pentanal, hexanal, 3-methyl-hexanal, heptanal, 3-methyl-2-butenal, 3,3-dimethyl-hexanal, nonanal, octanal, decanal, E-2-nonenal, E-2-hexenal, E,E-2,4-heptadienal, E-2-octenal, E,E-2,4-nonadienal, 2-undecanal, and E,E-2,4-decadienal	[82]
			HHP treatments greatly enhanced the headspace contents of aldehydes (↑ 13.6–100.2%)	
			HHP exerted the most significant effects on the aldehydes of GBR, and 11 aldehydes were altered after treatments (methacrolein, 2-methyl-propanal, butanal, 2-methyl-butanal, 3-methyl-butanal, pentanal, hexanal, 3-methyl-hexanal, heptanal, 3-methyl-2-butenal, 3,3-dimethyl-hexanal, nonanal)	
			Some minor aldehyde compounds were increased by HHP, including E-2-nonenal, E-2-hexenal, E,E-2,4-heptadienal, E-2-octenal, E,E-2,4-nonadienal, 2-undecenal, E,E-2,4-decadienal.	
Green Asparagus Juice	200, 400, and 600 MPa/10–20 min	Samples were stored at 4 °C for two days until the volatile analysis via GC-MS	22 aldehydes were identified in the samples: 3-Methyl-butanal, Pentanal, 2-Butenal, Hexanal, 2-Methyl-2-butenal, 2-Pentenal, 2-Methyl-2-pentenal, 2,4-Pentadienal, Heptanal, 2-Hexenal, Octanal, 2-Ethyl-2- hexenal, 2-Heptenal, Nonal, 2-Octenal, 2,4-Heptadienal, Decanal, Benzaldehyde, Trans-2-dodecen-1-al, Nonenal, 2,4-Octadienal, and 2,4-Decadienal	[84]
			Aldehydes were the main volatile compounds in green asparagus juice, which included hexanal, 2-heptenal, pentanal, 2-methyl-2-butenal, 2-butenal, 2-pentenal, 2-octenal, 2-hexenal, 3-methyl-butanal, and nonal	
			HHP treatments markedly (*p* < 0.05) increased the concentrations of aldehydes (↑ 25.3–66.1%)	
			The asparagus juice treated at 200 MPa for 20 min had the highest aldehydes concentrations (↑ 66.1%)	
Mulberry Juice	200, 300, 400, 500, and 600 MPa/10 min	Samples were incubated at 4 and 25 °C for 28 days after HPP treatments, the volatile analysis was made via GC-MS	13 aldehydes were identified in the samples: 3-Methylbutanal, Hexanal, 2,4-Pentadienal, Heptanal, 2-Hexenal, Octanal, (E)-2-Heptenal, Nonanal, Furfural, Benzaldehyde, (E)-2-Nonenal, (E,Z)-2-6-Nonadienal, and β-Cyclociral	[83]
			The overall content of aldehydes was enhanced after HHP processing (↑ 54.6%)	
			The concentration of (E)-2-Heptenal, benzaldehyde, and (E)-2-Nonenal decreased significantly (↓ 42.6–82.1%) after all HHP treatment conditions	
**Alcohols**				
Red Plum Puree	400 and 600 MPa/1 s, 2.5–5 min	Samples were stored at −80 °C for one week before volatile analysis via headspace SPME-GC	6 alcohols were found in the sample: Octan-1-ol, (Z)-Hex-3-en-1-ol, Hexan-1-ol, 2-Ethylhexan-1-ol, Oct-1-en-3-ol, and Non-1-en-3-ol	[43]
			Among the six alcohols isolated, three were modified by HHP (hex-3-en-1-ol, oct-1-en-3-ol, and non-1-en-3-ol). In general, a little decrease in the total AAU of alcohols was observed after HHP (↓ 4.1–12.2%)	
			Hexan-1-ol, which was the second most abundant contributor of the aroma of plum puree, was not modified after HHP, although a small decrease was observed in their levels after processing (↓ 4.2–9.9%)	
			A significant decrease in the level of Hexen-3-ol was found in the processed purees at less intense conditions (↓ 19.4% and 20.8% for 400 MPa, 1 s and 2.5 min respectively) with respect to the unprocessed puree	
			The overall content of alcohols decreased with HHP processing (4–12.2%). The largest decrease in concentration occurred at 400 MPa for 150 s.	
Keitt Mango Juice	200, 400, and 600 MPa/15 min	Samples were frozen in liquid nitrogen after processing and stored at −80 °C for two weeks until the volatile analysis via GC-MS	9 alcohols were identified in the samples: ethyl alcohol, 3-methyl-1-butanol, (Z)-2-penten-1-ol, (Z)-3-hexen-1-ol, 1-heptanol, (E)-3-hepten-1-ol, (Z)-3-nonen-1-ol, α-phellandren-8-ol, and (E,Z)-3,6-nonadien-1-ol	[85]
			Alcohols contributed to 5.1% of the quantitative volatile portion of the sample	
			C_9_ alcohols such as (E,Z)-3,6-nonadien-1-ol seemed to decrease with increase of processing pressure	
Hami Melon	400 and 500 MPa/10 min	Samples were stored at 4 °C overnight, the volatile analysis was made via GC-MS	5 alcohols were identified in the samples: Ethanol, (Z)-3-hexen-1-ol, Nonan-1-ol, (Z)-3-decen-1-ol, and Z)-6-nonen-1-ol	[87]
			Alcohols contributed around 5.9%–7.6% of the volatile portion of the sample	
			Samples exposed to HHP treatments showed evidently lower alcohol contents (↓ 50.8–90.9%)	
Green Asparagus Juice	200, 400, and 600 MPa/10–20 min	Samples were stored at 4 °C for two days until the volatile analysis via GC-MS	5 alcohols were identified in the samples: Geraniol, 3-Methyl-1,2-cyclopentanediol, 2-Methyl-2-undecanethiol, Nonanol, and 1-Octen-3-ol	[84]
			Alcohol concentration of green asparagus juice processed at 200 MPa was like that of the control (↓ 3.9%)	
			HHP at 400 and 600 MPa significantly (*p* < 0.05) decreased the alcohols concentration compared with the control (↓ 5.3% and 24.7%)	
Raw Goat Milk Cheese	400 and 600 MPa/7 min	Samples were stored at −80 °C after HPP processing until its analysis, which took place at room temperature (~25 °C) at Day 1, Day 30, and Day 60 (labeled Day 50 because of the maturation process) after treatment	16 alcohols were identified in the samples: 2-Propanol, Ethanol/2-methoxy, 2-Butanol 1-Propanol, 2-Pentanol, 1-Butanol, 3-Methyl-1-butanol, 1-Pentanol, 3-Buten-1-ol, 3-methyl, 2-Heptanol, 3-Penten-2-ol, 2-Nonen-1-ol, 2-Methyl-cyclohexanol, 2-Nonanol, 2-Furanmethanol, and 4-Butoxy-1-butanol	[88]
			On Day 1, the total concentration of alcohols fell by 63.6% and 72.6% for processing conditions of 400 and 600 MPa respectively	
			On Day 30, the total concentration of alcohols fell by 53.6% and 52.9% for processing conditions of 400 and 600 MPa respectively	
			On Day 50, the total concentration of alcohols fell by 23.6% and 31.2% for processing conditions of 400 and 600 MPa respectively	
			2-Nonanol, 2-Nonen-1-ol, and 3-Buten-1-ol, 3-methyl are the only 3 alcohols whose concentration seemed to increase, although very slightly, with HHP treatment conditions	
Mulberry Juice	200, 300, 400, 500, and 600 MPa/10 min	Samples were incubated at 4 and 25 °C for 28 days after HPP treatments, the volatile analysis was made via GC-MS	14 alcohols were identified in the samples: 1-Butanol, 1-Penten-3-ol, 1-Pentanol, 2-Ethyl-1-Butanol, 1-Hexanol, (Z)-4-Hexen-1-ol, (Z)-7-Tetradecen-1-ol, 1-Octen-3-ol, Linalool oxide, 1-Decanol, Linalool, 1-Octanol, Terpinen-4-ol, and 1-Nonanol	[83]
			HHP treatments enhanced the overall content of alcohols (↑ 46.5% at 500 MPa/10 min)	
			6 alcohols identified in the control samples were not found in HHP treated samples (2-Ethyl-1-Butanol, (Z)-7-Tetradecen-1-ol, 1-Octen-3-ol, 1-Decanol, 1-Octanol, and Terpinen-4-ol)	

SPME-GC, solid phase microextraction–gas chromatography; GC-MS, gas chromatography–mass spectrometry; ↓ = Decrease; ↑ = Increase.

### 3.2. HHP Effect on Ketones, Esters, and Lactones

An overview of the main changes in the concentration of ketones, esters, and lactones after HHP treatments at 100–800 MPa is shown in Table 3. On average, it seems that HHP treatments resulted in either an increment or a null effect on the total concentration of ketones with only one of the referenced matrices presenting an overall decrease in ketone concentration (kiwifruit pulp). The concentration of esters in most of the referenced matrices decreased when processed under HHP. Lactones remained mostly unaffected by HHP treatments below 400 MPa, and in one instance the overall content of lactone concentration was enhanced by HHP treatment, but, once the pressure reached and exceeded 500 MPa, the content of lactones decreased significantly with one exception that is discussed in a further paragraph. 

Ketones can be products of either lipid oxidation such as aldehydes or amino acid degradation induced by the Strecker reaction where an α-dicarbonyl compound reacts with an amino acid resulting in a carbonyl-amine condensation. Protein-deficient matrices such as Hami melons may remain unaffected to HHP treatments due to the lack of free amino acids necessary to form ketones [87], and in the specific case of this matrix, lipid oxidation mechanism produced aldehydes rather than ketones due to them being primary alcohols. Pressure level ranges also seem to play an important role in the overall effect HHP treatments have on ketone content. In Keitt mango juice, no significant difference on the concentration of β-ionone was found in treated samples, except at a pressure of 600 MPa [85]. Since in this matrix β-ionone is synthesized from carotenoids by dioxygenase, the performance of β-ionone could be attributed more to the behaviors of carotenoids rather than ketones. Other examples of specific pressure ranges affecting ketone contents are: red plum puree [43], where processing at 400 MPa for 1 s increased nonan-2-one content, while purees processed at 600 MPa for 1 and 150 s showed similar levels as unprocessed puree and therefore are considered unaffected, and kiwifruit pulp where HHP treatments at 400 MPa for 5 and 10 min significantly increased the total ketones content, while ketones concentration greatly decreased in the rest of HHP treatments [81]. In two different cultivars of brown rice, every HHP treatment greatly increased the headspace content of ketones [89]. Among the ketone species modified by HHP in this matrix, 2,3-butanedione was the most abundant ketone, this ketone had the largest increment at 300 MPa for *Indica* cultivars but at 500 MPa for *Japonica* cultivars, suggesting the presence of significant interactive effects between pressure and rice cultivars. The effect of HHP treatments on all ketones present on the samples of raw goat cheeses was statistically significant, both when applied at the beginning of the maturation process and at the end of the ripening [87]. Treatments applied at the beginning of maturation increased the levels of ketones, so the highest levels of 2-pentanone, 4-heptanone, 2-heptanone, 2-octanone, and 2-nonanone were found in cheeses treated at Day 1.

Changes in ketones due to HHP were not as intense when treatment was applied at the end of ripening but were still deemed to be statistically significant. The authors observed an increase in methyl ketones in goat cheese samples treated at the initial day of the maturation process and attributed it to the fact that ketones did not undergo reduction to alcohols, since lower levels of alcohols were found in HHP-treated cheeses than control ones. It is also inferred that HHP could facilitate the production of methyl ketones from free fatty acids. In green asparagus juice, HHP treatments maintained a higher ketone concentration when compared to traditional thermal treatments [84].

Esters hydrolyze to their respective acids and/or alcohols during HHP treatment, the tendency of esters to decrease during processing can be explained based on a possible hydrolytic effect that HHP causes in the matrices. For red plum puree the levels of most esters present in the matrix decreased during the HHP treatments only (Z)-hex-3-enyl acetate, quantitatively the major ester present, and hex-3-enyl benzoate were not modified after HHP processing, preserving the original aroma of puree [43]. Some esters such as hex-2-enyl acetate and ethyl decanoate decreased at the less intense treatment conditions but increased when the highest pressures and holding times, 600 MPa/300 s, were applied. For Keitt mango juice, the overall concentration of esters was found to decrease after HHP treatments [85]. The most likely precursors for the esters in this matrix were lipids and amino acids; the key enzyme in the rate-limiting step for ester biosynthesis was alcohol acyltransferase enzyme, whose activity seemed to decrease as the pressure increased. Out of the four ester species originally presented in mulberry juice, two were only found in HHP and thermal treated samples and not in control samples, octyl formate and γ-nonanolactone; γ-unsecalactone was not found in any of the HHP-treated samples; and the remaining ester, butyl acetate, had a significantly reduced concentration when compared to control samples [83]. For Hami melon, pressure treatments caused the disappearance of six esters [33]. When compared to the control, ethyl 2-methylbutanoate, 2,3-butanediol diacetate, ethyl acetate, and propyl acetate were all increased significantly by HHP treatments; no overt difference was noticed between the concentrations at 400 and 500 MPa processing conditions. A tendency that esters presented when submitted to HHP treatments is that the most intense treatments, above 500 MPa, had the best effect to preserve aroma compounds.

Lactones, being a type of ester, share many similarities to the behavior explained in the previous paragraph, but, due to their cyclic nature, there are some properties that set them apart. While ester concentration tends to decrease when submitted to HHP treatments, lactones either increased or remained unaffected after the treatments. Only one matrix presented a decrease in the overall concentration. In red plum puree, only one lactone was identified in the samples, γ-octalactone [43]. No significant difference was found between fresh and HHP treated with 400 MP, but, at 600 MPa, a decrease in concentration was observed. A similar case can be seen in raw goat milk cheese, where one lactone was identified in the samples, δ-decalactone [88]. HHP treatments enhanced the content of δ-decalactone found in the samples, with the greatest of these increases found in samples treated by 400 MPa on Day 50. In human breast milk, another single lactone was found in the samples, γ-crotonolactone [90]. Except for 400 MPa/3 min, where the content decreased, HHP treatments enhanced the overall content of γ-crotonolactone found in the samples. Two lactones were identified in rice wine samples, γ- butyrolactone and γ-nonalactone [91]. No significant increase was identified since the concentrations of the two lactones slightly increased in both the control and HHP-treated samples. Strawberry was the only matrix where the concentration of lactones significantly was reduced after HHP treatments, with one lactone (γ-decalacton) identified in the samples [92]. The concentration of γ-decalactone was not affected by pressure treatments at 200 MPa, but significant decreases in concentration were found significant for pressure-treatment at both 500 and 800 MPa. Overall, lactones contradict the tendency presented by esters; an increase in their concentrations can be found at lower pressure ranges (200–400 MPa), while more intense treatment conditions (above 500 MPa) result in a decrease of lactone concentration.

**Table 3:** Main changes in the concentration of ketones, esters, and lactones after HHP treatments.

Matrix	Processing Conditions	Storage Conditions and Technique of Analyses	Main Results	Reference
**Ketones**				
Red Plum Puree	400 and 600 MPa/1 s, 2.5–5 min	Samples were stored at −80 °C for one week before volatile analysis via headspace SPME-GC	2 ketones were found in the samples: 5-Hepten-2-one·6-methyl-one and Nonan-2-one	[43]
			The ketones content was low in the purees, accounting for less than0.3% of the total volatiles	
			Nonan-2-one content was affected by HHP (↑ 7–43.4%) while 6-methyl-5-hepten-2-one content remained unchanged	
			Processing at 400 MPa/1 s increased nonan-2-one content (↑17.9–43.4%), while purees processed at 600 MPa for 1 and 150 s showed similar levels as unprocessed puree	
Kiwifruit Pulp	400 and 600 MPa/5–15 min	Samples were stored in the dark at 4 °C for 40 days before volatile analysis via SPME and GC-MS	10 ketones were identified in the samples: 3-Hexanone, 2-Hexanone, 2-Pentanone, 1-Penten-3-one, 1-Octen-3-one, 2,5-Hexanedione, 3-Octanone, 3-Pentanone, 3-Heptanone, and 2-Heptanone	[81]
			HHP treatments (400 MPa for 5 min/10 min) significantly increased the total ketones content (↑ 21.5–2255.4%) while a decrease of ketones concentration was detected in the rest of HHP treatments (↓ 8.7–97.2%)	
			The total content of ketones in pulp beverage processed at 500 MPa for 10 min increased steadily and remained stable over 20 days, which revealed that HHP at 500 MPa for 10 min was more conducive to the retention of ketones than HT	
Keitt Mango Juice	200, 400, and 600 MPa/15 min	Samples were frozen in liquid nitrogen after processing and stored at −80 °C for two weeks until the volatile analysis via GC-MS	Only one ketone was identified in the samples, β-ionone	[85]
			HHP treatment showed no significant difference on the concentration of β-ionone	
Hami Melon	400 and 500 MPa/10 min	Samples were stored at 4 °C overnight, the volatile analysis was made via GC-MS	2 ketones were identified in the samples: (5E)-6,10-dimethylundeca-5,9- dien-2-one and 2,2,6-trimethyl-3-butanedione	[87]
			No significant differences could be seen in the ketones between HHP and untreated samples were observed	
			Ketones accounts for around 0.4% of the total volatiles, making it the lowest represented functional group of the matrix	
Germinated Brown Rice (GBR)	100, 300 and 500 MPa/15 min	Samples were stored at 4 °C for 36 h until the volatile analysis via GC-MS	12 ketones were identified in the samples: 2-butanone, 2,3-butadione, 3-penten-2-one, 2-methyl-3-pentanone, 4,4-dimethyl-2-cyclopenten-1-one, 1-cyclopropyl-1-propanone, 3,4,5-trimethyl-2-cyclopenten-1-one, 2-heptanone, 2-hydroxy-3-butanone, 2-nonanone, 3-octen-2-one, 6-methyl-2-heptanone, 5-pentyl-2-(3H)-dihydro-furanone	[82]
			HHP treatments greatly enhanced the headspace contents of ketones (↑ 35.7–1832.6%)	
			Among the ketones modified by HHP, 2,3-butanedione with a fruitynote was the most abundant ketone, which had the largest increment at 300 MPa for DHX cultivars but at 500 MPa for SQD cultivars	
Green Asparagus Juice	200, 400 and 600 MPa/10–20 min	Samples were stored at 4 °C for two days until the volatile analysis via GC-MS	8 ketones were identified in the samples: 3-Octanone, 1-Octen-3-one, 6-Methyl-5-hepten-2-one, 3-Undecen-2-one, 3-Octen-2-one, 3,5-Octadien-2-one, Geranyl acetone, and β-ionone	[84]
			HHP treatments maintained higher ketones concentrations than thermal treatment	
			1-Octen-3-one and β-ionone were the only ketones whose concentration decreased after HHP processing, the greatest decrease occurring at 400 MPa at 10 min (↓ 28.9%) and 400 and 600 MPa at 10 min (↓ 35.7%) respectively	
			6-Methyl-5-hepten-2-one presented the largest increase in concentration amongst the ketone species at 200 MPa/10 min processing conditions (↑ 400%)	
Raw Goat Milk Cheese	400 and 600 MPa/7 min	Samples were stored at −80 °C after HPP processing until its analysis. Volatile analysis occurred at Day 1, Day 30, and Day 60 (labeled Day 50 because of the maturation process) after treatment.	8 ketones were identified in the samples: 2-Butanone, 2-Pentanone, 4-Heptanone, 2-Heptanone, 2-Octanone, Cyclohexanone, 2-methyl, 2-Nonanone, and 2-Undecanone	[88]
			HHP treatment was applied at three different stages of ripening, Day 1, 30 and 50 of the maturation processes.	
			The total amount of ketones increased (↑ 123.2–162.1%) after HHP on Day 1	
			The total amount of ketones increased (↑ 42.4–63%) after HHP on Day 30	
			The total amount of ketones decreased (↓ 25.6–46.4%) after HHP on Day 50.	
Mulberry Juice	200, 300, 400, 500, and 600 MPa/10 min	Samples were incubated at 4 and 25 °C for 28 days after HPP treatments, the volatile analysis was made via GC-MS	6 ketones were identified in the samples: 3-Octanone, 4-Octen-3-one, 6-Methyl-5-hepten-2-one, 1-Hexyn-3-one, β-Damascenone, and β-Ionone	[83]
			HHP processing enhanced the overall content of ketones (↑ 13.2–197.4%) except for 3-Octanone which was not present in HHP treated samples	
**Esters**				
Red Plum Puree	400 and 600 MPa/1 s, 2.5–5 min	Samples were stored at −80 °C for one week before volatile analysis via headspace SPME-GC	11 types of esters were isolated: Ethyl acetate, Hexyl acetate, [(Z)-Hex-3-enyl] acetate, [(E)-Hex-2-enyl] acetate, Pentyl acetate, [(Z)-Hex-3-enyl] butanoate, Hexyl butanoate, [(Z)-Hex-3-enyl] hexanoate, Hexyl hexanoate, [(E)-Hex-3-enyl] benzoate, and Ethyl decanoate	[43]
			(Z)-hex-3-enyl acetate was quantitatively the major ester isolated (↓ 1.5–11.9% after processing)	
			Esters were the family of compounds most affected by HHP. The levels of most esters slightly decreased after the treatments (↓ 6.1–17.2%)	
Keitt Mango Juice	200, 400, and 600 MPa/15 min	Samples were frozen in liquid nitrogen after processing and stored at -80 °C for two weeks until the volatile analysis via GC-MS	4 esters were identified in the samples: ethyl acetate, ethyl butyrate, nonyl acetate, and γ-octalactone	[85]
			Esters contributed to 4.9% of the total content of volatile compounds	
			Concentrations of esters, were found to reduce after HHP processing	
Hami Melon	400 and 500 MPa/10 min	Samples were stored at 4 °C overnight, the volatile analysis was made via GC-MS	23 esters were identified in the samples: Methyl acetate, Ethyl acetate, Propyl acetate, Methyl butyrate, Ethyl 2-methylpropanoate, 2-methyl propyl acetate, Methyl 2-methylbutyrate, Ethyl butanoate, Methyl valerate, Ethyl 2-methylbutanoate, 2-methyl butyl acetate, Methyl ethyl thioacetate, Ethyl caproate, 3-hexenol acetate, Hexyl acetate, 2,3-butanediol diacetate, 2-butanol-2 methyl acetate, Heptyl acetate, Methyl phenylacetate, Dimethyl 2-methylpropionate, Butyl butyrate, Diethyl phthalate, and Isopropyl palmitate	[87]
			Esters contributed 59.6%–71.3% of the total volatile compounds of the 400 and 500 MPa samples, respectively	
			Pressure levels caused the disappearance of six esters (methyl acetate, methyl butyrate, methyl 2-methylbutyrate, methyl valerate, heptyl acetate, and isopropyl palmitate)	
			Compared with the control, ethyl 2-methylbutanoate, 2,3-butanediol diacetate, ethyl acetate, and propyl acetate were increased significantly (↑ 8.9–172%) by HHP, whereas no overt difference was noticed between 400 and 500 MPa	
Mulberry Juice	200, 300, 400, 500, and 600 MPa/10 min	Samples were incubated at 28 °C after HPP treatments, the volatile analysis was made via GC-MS	4 esters were identified in the samples: Butyl acetate, Octyl formate, γ-Unsecalactone, and γ-Nonanolactone	[83]
			Octyl formate and γ- Nonanolactone were not present the control samples and only were found in treated (HHP and thermal) samples	
			γ-Unsecalactone was not found in HHP treated samples	
			Butyl acetate had a reduced concentration (↓ 10.9%) in HHP treated samples	
**Lactones**				
Red Plum Puree	400 and 600 MPa/1 s, 2.5–5 min	Samples were stored at −80 °C for one week before volatile analysis via headspace SPME-GC	One lactone was identified in the samples: Furan 2-ethyl-one	[43]
			Furan 2-ethyl-one contributed only 0.4–0.5% of total volatiles	
			Furan 2-ethyl decreased (↓ 18.2–31.8%) after some HHP conditions (400 MPa/1 s and 600 MPa/150 s)	
Keitt Mango Juice	200, 400, and 600 MPa/15 min	Samples were frozen in liquid nitrogen after processing and stored at −80 °C for two weeks until the volatile analysis via GC-MS	One lactone was identified in the samples: γ-octalactone	[85]
			No significance difference was found between fresh and HHP with 400 MPa for the concentration of γ-octalactone, but at 600 MPa a decrease in concentration was observed	
Strawberry Coulis	200, 500 and 800 MPa/20 min	After HPP treatments, samples were stored and frozen at −18 °C until the extraction of volatile compounds which occurred seven days later	One lactone appears only in samples treated with HHP: γ-Decalactone	[92]
			The concentration of γ-Decalactone is not significantly affected by pressure treatments at 200 MPa (↓ 9.3%), but the decrease in concentration is significant for pressure-treatments of 500 and 800 MPa (↓ 87.8% and 47.8% respectively)	
Raw Goat Milk Cheese	400 and 600 MPa/7 min	Samples were stored at −80 °C after HPP processing until its analysis. Volatile analysis occurred at Day 1, Day 30, and Day 60 (labeled Day 50 because of the maturation process) after treatment	One lactone was identified in the samples: δ-decalactone	[88]
			HHP treatment enhanced (↑ 14.5–29% on Day 1) the content of δ-decalactone in the samples	
			The greatest content of δ-decalactone was found in samples treated by 400 MPa on Day 50 (↑ 61.8%)	
Human Breast Milk	400 and 600 MPa/3–6 min	Samples were stored at −80 °C until the volatile analysis took place, which was carried out within one month after processing	One lactone was found in the samples: γ-Crotonolactone	[90]
			Other than 400 MPa for 3 min (↓ 33.3%), all other HHP treatments enhanced the overall content of γ-Crotonolactone in the samples (↑ 190.6–2104.8%)	
Hongqu Rice Wine	200 MPa and 550 MPa/30 min	HPP treated samples were stored at 10–15 °C for 18 months after treatment to allow the fermentation process to take place	Two lactones were identified in the samples: γ- butyrolactone and γ-nonalactone	[91]
			The levels of lactones increased slightly in both control and HHP-treated wine samples during storage in pottery; however, during aging, no significant differences were observed	

SPME-GC, solid phase microextraction–gas chromatography; GC-MS, gas chromatography–mass spectrometry; ↓ = Decrease; ↑ = Increase.

### 3.3. HPP Effects on Terpenes and Phenols

An overview of the main changes in the concentration of terpenes and phenols after HHP treatments at 100–600 MPa is shown in Table 4. There does not seem to be a clear tendency in the concentration of terpenes after HHP processing; there are matrices where the content of terpene species remains unaffected, others where it increases, and some where terpene content drops entirely. 

For red plum puree, out of the four terpenes identified in the samples (β-Cyclocitral, β-ionone, *trans*-Geranylacetone, and β-Damascenone), only β-ionone had its concentration significantly altered by HHP treatments, demonstrating a more noticeable increase at 600 MPa processing conditions [43]. In the case of human breast milk, out of the two terpenes identified in the untreated samples (α-Pinene and D-Limonene), HHP seemed to totally decrease the concentration of α-Pinene since it was not found in either processing condition [90]. At 600 MPa for 3 min, D-Limonene appeared to slightly increase its concentration, while at all other processing conditions resulted in a significant decrement. 

The only matrix in which all terpenes appeared to be positively influenced by HHP treatments among the referenced studies can be found in raw goat milk cheese, where limonene concentration, the only terpene identified in the samples, increased in both 400 and 600 MPa processing conditions [88]. 

The most likely explanation for the variability in effects after treatment is attributed to enzymatic reactions; since HHP is capable of both enhancing and inhibiting enzymatic activity depending on its structure [73], it can be inferred that oxidation/degradation reactions involving terpenes can only occur over suitable pressure ranges. It is also worth mentioning that, with the exception of mango juice, HHP appeared to have less influence on terpene concentration compared to thermal processing due to the latter being responsible for the degradation of terpenes [85,90]. 

Phenol concentration appears to follow the same tendency as alcohols did under HHP treatments, with most matrices presenting a decrease in concentration levels except for cooked rice whose phenol content appeared to increase when compared to untreated samples [93]. The only phenol compound identified in green asparagus juice was 2,4-Di-tert-amyl phenol, which decreased significantly with all three processing conditions with most significant decrease occurring at 400 MPa for 10 min [84]. Out of the three phenolic compounds identified in brown rice samples, phenol, 2,4-Di-tert-butylphenol, and 4-Vinylguaiacol, only the concentrations of phenol at all three HHP processing conditions and 4-Vinylguaiacol at 500 MPa had their concentrations increased; 2,4-Di-tert-butylphenol and both other processing conditions (100 and 300 MPa) for 4-Vinylguaiacol reduced concentrations after HHP treatment [88]. Only two phenol compounds were identified in cow milk samples, and one of them, 3-methylphenol, was not identified in any of the HHP treated samples, while the other phenolic compound, 2,4-bis(1,1-dimethylethyl)-phenol, decreased in concentration after all processing conditions. 

The concentration of phenolic compounds in all matrices is attributed to the activity of polyphenol oxidase (PPO); if this enzyme is enhanced, the concentration of phenolic compounds is decreased, and, if it is inhibited, the opposite occurs. The concentration of phenolic compounds was found to be greater in thermally treated samples than in HHP samples [83,94], likely because PPO presents a greater degree of inhibition when submitted to thermal treatments than HHP ones. 

**Table 4:** Main changes in the concentration of terpenes and phenols after HHP treatments.

Matrix	Processing Conditions	Storage Conditions and Technique of Analyses	Main Results	Reference
**Terpenes**				
Red Plum Puree	400 and 600 MPa/1 s, 2.5–5 min	Samples were stored at −80 °C for one week before volatile analysis via headspace SPME-GC	4 terpenes were identified in the samples: β-Cyclocitral, β-ionone, trans-Geranylacetone, and β-Damascenone	[43]
			The total content of terpenes accounted for 0.3–0.5% of total volatiles	
			Only β-ionone was affected by HHP (↑ 43.7–86%) while the other three compounds remained unaffected	
Keitt Mango Juice	200, 400, and 600 MPa/15 min	Samples were frozen in liquid nitrogen after processing and stored at −80 °C for two weeks until the volatile analysis via GC-MS	10 terpenes were identified in the samples: α-pinene, 3-carene, β-myrcene, δ-limonene, β-phellandrene, γ-terpinene, (E)-β-ocimene, terpinolene, β-caryophyllene, and α-caryophyllene	[85]
			The concentrations of 3-carene, β-myrcene, and γ-terpinene undergoing HHP processing exhibited different characteristics, which kept increasing up to the pressure of 400 MPa and decreased above 400 MPa	
			HHP appeared to have less influence on terpenes compared to heating	
Raw Goat Milk Cheese	400 and 600 MPa/7 min	Samples were stored at −80 °C after HPP processing until its analysis. Volatile analysis occurred at Day 1, Day 30, and Day 60 (labeled Day 50 because of the maturation process) after treatment	One terpene was found in the samples: limonene	[88]
			The overall content of limonene in the samples was enhanced by HHP treatments (↑ 340–680% on Day 1)	
			The greatest content of limonene was found in samples treated by 400 MPa on Day 30 (↑ 740%)	
Human Breast Milk	400 and 600 MPa/3–6 min	Samples were stored at −80 °C until the volatile analysis took place, which was carried out within one month after processing	Two terpenes were identified in the samples: α-Pinene and D-Limonene	[90]
			α-Pinene was not found in any of the HHP treated samples (↓ 100%)	
			Other than 600 MPa for 3 min (↑ 4.8%), HHP treatments seemed to decrease the levels of D-Limonene in the samples (↓ 5.2–18.8%)	
**Phenols**				
Green Asparagus Juice	200, 400 and 600 MPa/10–20 min	Samples were stored at 4 °C for two days until the volatile analysis via GC-MS	One phenol was identified in the samples: 2,4-Di-tert-amyl phenol	[84]
			Concentration of 2,4-Di-tert-amyl phenol decreased significantly (↓ 41.9–72.1%)	
Germinated Brown Rice (GBR)	100, 300 and 500 MPa/15 min	Samples were stored at 4 °C for 36 h until the volatile analysis via GC-MS	Three phenols were identified in the samples: Phenol, 4-ethyl-phenol, and 4-Vinylguaiacol (2-methoxy-4-vinylphenol)	[82]
			Phenol was the only compound whose concentration increased with HHP treatments, the greatest and lesser increase occurred at 500 MPa (↑ 258.4%) and 100 MPa (↑ 27.4%) respectively	
			4-ethyl-phenol appeared to decrease slightly in all processing conditions; ↓ 12.3%, ↓ 6.2%, and ↓ 11.1% for 100, 300, and 500 MPa respectively	
			Except for 500 MPa (↑ 16.7%), concentration of 4-Vinylguaiacol decreased with HHP treatments; ↓ 25% and ↓ 16.7% for 100 and 300 MPa respectively	
Cooked Rice (Wuchang and Complete Wheel)	200, 400 and 600 MPa/10 min	Samples were stored at 4 °C for 1 h until the volatile analysis GC-MS	Two phenols were identified in the samples: 2-Methoxy-4-vinylphenol and Phenol, 2,4-bis (1,1-dimethylethyl)	[93]
			For both Whuchang and Complete Wheel rice all processing conditions led to an increase in 2-Methoxy-4-vinylphenol (↑ 29.8–64.7% and 33.9–173.2% respectively)	
			For Whuchang rice all processing conditions led to a decrease in Phenol, 2,4-bis (1,1-dimethylethyl) (↓ 23.8–43.7%) except for 400 MPa which increased its concentration (↑ 12.4%)	
			For Complete Wheel rice only 200 MPa processing conditions led to a decrease in Phenol, 2,4-bis (1,1-dimethylethyl) (↓ 5.7%) all other processing conditions increased its concentration (↑ 42.4–62.6%)	
Cow Milk	200, 400 and 600 MPa/1 × 5 min and 2 × 2.5 min	Samples were analyzed immediately after HPP treatments via GC-MS	Two phenol compounds were identified in the samples: 3-methylphenol and 2,4-bis(1,1-dimethylethyl)-phenol	[94]
			3-methylphenol was not found in any of the HHP treated samples	
			The overall content of 2,4-bis(1,1-dimethylethyl)-phenol decreased in HHP treated samples, the greatest and lesser decrease of content occurring at 200 MPa 2 × 2.5 min (↓ 77.6%) and 600 MPa 1 × 5 min (↓ 55.7%) respectively	

SPME-GC, solid phase microextraction–gas chromatography; GC-MS, gas chromatography–mass spectrometry; ↓ = Decrease; ↑ = Increase.

## 4. Final Remarks

HHP can both enhance and inhibit chemical reactions occurring in the matrices being subjected, and the consequences it can have on enzymes responsible for these reactions can be both desired and undesired, thus making HHP processing stand out as a unique alternative to conventional thermal processes. Given that many studied samples were stored at refrigerated or room temperature before volatile analysis, it is inferred that the chemistry regarding enzymes responsible for the described processes was affected during this time resulting in the modifications of the concentration of some aroma compounds. Aldehydes and ketones predominantly increased their levels after treatment, probably due to the oxidative processes of alcohols and phenols, which explains the decrease of alcohols and phenols when subjected to HHP. Esters and lactones concentration levels seemed to decrease due to hydrolysis reactions occurring after treatments, and terpene concentration showed mixed results due to the effects that HHP had on enzymes responsible for oxidation and reduction processes. The information presented in this article agrees that HHP treatment in certain processing conditions can preserve select volatile aroma compounds more effectively than thermal processing, proving HHP is an interesting alternative technology as a widespread process that warrants further research that allows both the optimization and control the effect of HHP so that it would be possible to make accurate predictions of the effect it will have on the subjected matrix.
